# The New Precision Stewards?

**DOI:** 10.3390/jpm12081308

**Published:** 2022-08-12

**Authors:** Karen M. Meagher, Sara Watson, Gina A. Suh, Abinash Virk

**Affiliations:** 1 Biomedical Ethics Research Program, Mayo Clinic, Rochester, MN 55905, USA; 2 Division of Public Health, Infectious Disease, and Occupational Medicine, Mayo Clinic, Rochester, MN 55905, USA

**Keywords:** antimicrobial drug resistance, microbial genetics, antimicrobial stewardship, biomedical ethics, biomedical research, environment and public health

## Abstract

The precision health era is likely to reduce and respond to antimicrobial resistance (AMR). Our stewardship and precision efforts share terminology, seeking to deliver the “right drug, at the right dose, at the right time.” Already, rapid diagnostic testing, phylogenetic surveillance, and real-time outbreak response provide just a few examples of molecular advances we dub “precision stewardship.” However, the AMR causal factors range from the molecular to that of global health policy. Mirroring the cross-sectoral nature of AMR science, the research addressing the ethical, legal and social implications (ELSI) of AMR ranges across academic scholarship. As the rise of AMR is accompanied by an escalating sense of its moral and social significance, what is needed is a parallel field of study. In this paper, we offer a gap analysis of this terrain, or an agenda for “the ELSI of precision stewardship.” In the first section, we discuss the accomplishments of a multi-decade U.S. national investment in ELSI research attending to the advances in human genetics. In the next section, we provide an overview of distinct ELSI topics pertinent to AMR. The distinctiveness of an ELSI agenda for precision stewardship suggests new opportunities for collaboration to build the stewardship teams of the future.

## 1. Introduction

Does precision health encompass the advancement in mitigating the rise of antimicrobial resistance? Specifically, antimicrobial resistance (AMR) is a compelling candidate for the advancement of molecular technologies, including genomics. At the genetic level, variants that render bacteria, viruses, and fungi capable of withstanding some treatments have been dubbed the “resistome”. On top of the burdens of infection, resistance can thwart access to effective treatment. In this sense, the promissory nature of the genomic era shares a vision of precision with stewardship, to deliver “the right drug, at the right time, at the right dose” [1,2,3,4]. Already, rapid diagnostic testing, phylogenetic surveillance, and real-time outbreak response provide just a few examples of molecular advances we dub “precision stewardship”.

At the same time, the COVID-19 pandemic has also demonstrated the folly of neglecting social dynamics around infectious diseases. An emerging consensus states that AMR is best understood as a microbial and social problem. The result has been ongoing calls for increased investment in AMR social science, both within the United States (U.S.) and globally [5,6,7,8,9,10]. To these important calls, we highlight how the rise of AMR is increasingly depicted in moral terms [11,12,13,14,15]. Documenting this trend of increasing moral underpinnings in antimicrobial (AM) stewardship, Dyar et al. note how “the focus has moved away from technical descriptions (drug, dose, duration, etc.), towards concepts of responsibility” [16]. AMR has been framed variously (Table 1) as a conflict between patients’ short-term and long-term interests [17], a failure of the health systems to prioritize prevention [18], a challenge of collective action and solidarity [19,20], and in terms of One Health policy encompassing interlocking responsibility for environmental, animal, and human health [21,22].

Mirroring the cross-sectoral nature of the AMR literature, research addressing the ethical, legal, and social aspects of AMR remain scattered across wide-ranging areas of scholarship [27]. The social science on AMR has lagged behind that of other infectious diseases [28], but has increased since 2016 [27]. If the rise of AMR is indeed accompanied by an escalating sense of its moral importance, dynamic social contexts, and ongoing policy questions, what is then needed is a parallel field of study. One place to look for examples of such collaborations is the Ethical, Legal, and Social Implications (ELSI) Program of the National Human Genome Institute (NHGRI). For example, NHGRI previously supported multidisciplinary research in conjunction with the Human Microbiome Project [29]. To further explicate the current state of knowledge for AMR, in this paper we offer a gap analysis of this terrain, or an agenda for “the ELSI of precision stewardship.” In the first section, we discuss some of the main foci of ELSI research in human genetics that are likely to be relevant as microbial genetics also advances. In the next sections, we provide an overview of distinct ELSI topics pertinent to AMR, including its situatedness in the context of infection prevention and control (IPC) and stewardship.

## 2. Lessons Learned: ELSI and Precision Medicine

### 2.1. A National Investment in Multidisciplinary Inquiry

The NHGRI ELSI program was infamously borne of comments made by James Watson during a 1988 press conference announcing his leadership of the Human Genome Project [30]. The Human Genome Project was a U.S. research initiative tasked to grow our understanding of genetic inheritance by supporting international research collaborations to sequence and map the human genome. Belying his own biases on race and sex, Watson announced a dedicated line of funding for supporting social science and humanities inquiry to help counter the legacy of eugenics. Thus, the 2–3% intramural budget for the NHGRI ELSI program was formed and has been sustained for half a century. The early decades of ELSI research paralleled genetic advances, focusing on familial and social experiences accompanying highly heritable Mendelian traits. More contemporary ELSI research continues to accompany geneticists’ efforts to capture more complex heritable conditions, such as through polygenic risk scores that reflect the additive contribution of many genes to chronic disease risk, including cardiac conditions and diabetes. It is beyond the scope of this article to describe the full breadth of human genetics ELSI scholarship; the ELSIhub online platform, however, has set out to provide a curated set of overviews to increase the impact of this broadly multidisciplinary field of study [31]. In this section, we highlight three domains of human genetics ELSI research that offer lessons for a parallel research agenda for AMR ELSI (Table 2).

### 2.2. Assessing Understanding and Attitudes

#### 2.2.1. Human Genomics

A great deal of ELSI research effort has been directed toward ascertaining dynamic public attitudes toward advances in human genetics [32,33]. Ethical concerns about genetics include worries that social attitudes might lean inaccurately toward genetic determinism; the belief that the genes are always strongly causal of health or behavioral outcomes. Determinist beliefs are especially worrisome if they generate mistaken fatalism, e.g., “genetics is destiny,” or conflate sociocultural categories of race with biological differences [34]; cf. [35]. As genetic information became more available, attention to patients’ informational needs and health skills in relation to genetic information developed into the literature on genetic literacy. Genetic literacy scales have been developed and used to explore both conceptual and informational skills in both providers and patients [36,37,38]. Key findings include the need to improve genetic literacy to support successful clinical translation. In addition, ELSI scholars have also established frameworks for supporting decision making around disclosure of genomic research findings information uncovered in genetic research that is not always the primary object of study, but is nonetheless relevant to the genetic contributor’s health [39].

#### 2.2.2. Pathogen Genomics

For AMR, public attitudes research has explored the knowledge of AMR and the attitudes toward AM use [40]. Other studies in the U.S. have focused on the attitudes of subpopulations of interest due to health disparities in AM access and use, including Latinx populations [41], farmers [42], and parents [43]. Well-established implementation barriers to AM stewardship have also directed attention to attitudes among clinical professionals including physicians [44], nurses [45], ambulatory care physicians [46], pharmacists [47], and trainees [48]. Meanwhile, the occurrence of AMR infections overlaps with stakeholder experiences of hospital safety. Public awareness of hospital infections and valuation of cleanliness continues to raise concerns that data about hospital-acquired infections bring reputational harm to health systems and clinicians [49]. Importantly, patients are also increasingly recognized as another point of intervention for ensuring patient safety, especially in hand hygiene [50].

### 2.3. Health Equity

#### 2.3.1. Human Genomics

Human genetic research takes place in the long shadow of eugenics, with ethicists seeking to provide lessons on how to avoid and actively undermine the racism and ablism of past policy as genetic research advances [51,52]. ELSI discussions of injustice and equity began with providing legal protections, including through the Genetic Information Nondiscrimination Act and preventing employer genetic-based discrimination [53]. ELSI research has also identified health disparities in access to genetic testing and identified public concerns about the cost of genetic technologies along with longstanding barriers in access to clinical care [54,55,56]. Such research highlights the promissory nature of precision medicine, including the hope that its basis in population-based stratification can help direct interventions toward those most at risk [57].

ELSI scholars have often critically evaluated the ability of genomic science to deliver on the reduction of health inequities without a robust social policy companion. Human geneticists working in conjunction with ELSI scholars continue to grapple with how to appropriately disentangle social categories of race and ancestry genetics, a challenge that could exacerbate rather than redress inequity by reifying race as a biological construct [58]. These conversations are related to—but distinct from—concerns about diversity and representativeness within genetic datasets, which have often overrepresented individuals of European ancestry [59]. Precision medicine has also sought to elevate the role of patient empowerment; ELSI scholars have often sought to evaluate this potential promise, articulating the strengths, but also limitations, of health information in increasing patient advocacy, choice, and ability to address unmet health needs [60,61]. In doing so, the ELSI community has often sought to differentiate the hype and hope of emerging technologies, recognizing the social and political appeal of technological imperatives and incorporating anticipatory design insights from science and technology [62,63,64]. Overall, the health equity research portfolio seeks to ensure that no one is left behind as genomic research and technologies advance.

#### 2.3.2. Pathogen Genomics

Research on AMR equity and justice can take a leaf from the human ELSI playbook. Lack of access to basic medical care is inextricably linked to widely available over-the-counter antibiotics [65,66,67]. For conditions that disproportionately affect those in or from resource-poor settings, it will be yet more important to anticipate the need for research designs that enable participation of those often left out of biomedical research. The advent of mRNA COVID-19 vaccines shows us the potential of molecular genomics in facilitating unprecedented innovation to mitigate infectious diseases—but also the challenges in rendering these innovations widely accessible for global health [68]. Similarly, the disrupted AM drug development pipeline suggests echoes of human genetic ELSI work reflecting public concerns over genomic intellectual property and employment discrimination [69,70].

Another avenue to advance inquiry of how pathogen genomics and molecular epidemiology can advance equitably can start with regional and global distribution of laboratory and scientific capacity. Already, genomic surveillance activities are dramatically different depending on geography. Throughout global health, “helicopter” research has been critiqued for recapitulating colonial relationships, especially in access to high-quality research facilities and training. Laboratory capacity distribution is inextricably linked to efforts to increase personnel diversity, equity, and inclusion in the fields of microbiology, pathology, computational biology, pharmacy, and infectious disease. Places with innovative capacity will provide the training grounds for team science increasingly characteristic of largescale global collaboration.

### 2.4. Data Governance

#### 2.4.1. Human Genomics

The genetic revolution coincides with the era of big data. The insights possible from large data sets, including genome-wide association studies (GWAS), revealed heritable aspects of many conditions, including rare diseases. Questions of data governance from ELSI scholars accompanied these scientific advances. Among them, the increasing use of biospecimens in research led to examining whether broad consent to unspecified future research use was consistent with the ethical and regulatory standard of voluntary and informed consent. And while a full genetic sequence is often distinct to individuals, the common genes among blood relatives has challenged privacy norms and regulations governing the treatment of health information, as a single test result can bear implications for the health of third parties.

As human genetics has moved from bench to bedside so, too, has ELSI research. The ability of genetic test results to inform patient care has led to in-depth social science analyses about processes of variant interpretation (e.g., as pathogenic, likely pathogenic) which link to clinical actionability [71]. Included in this discourse are debates about when genetic test results are sufficient for clinical diagnosis and/or treatment, in comparison to phenotypic or other molecular biomarker assays that serve as proxies for pathogenesis. ELSI scholars have also identified the need for the translation of genetic results from research to clinical contexts, as well as the need to develop disclosure and clinical pathways that support practitioners at the forefront of patient care and genetic translation.

#### 2.4.2. Pathogen Genomics

Many of these same issues will impact the advancing science of AMR. Currently, governance discourse in largescale pathogen datasets is largely focused on scale and utility. Experts in resistome science and data curation appreciate its potential to improve stewardship efforts, and the need for an infrastructure that supports data-sharing. McArthur and Wright (2015) grapple with the phenotype-genotype gap if molecular epidemiology is to replace traditional susceptibility testing [72]. They attended to these considerations in generating the Comprehensive Antibiotic Resistance Database (CARD), a collection of AM resistance determinants that (1) supports research on the genetic underpinnings of resistant infections and (2) anticipates their clinical and public health use [73]. Other largescale datasets, such as ARG-ANNOT and ResFinder, also grapple with ways to maximize utility to the research community, a highly technical and dynamic challenge given the speed of vertical and horizontal genetic transfer [74,75].

ELSI research in biorepositories for human samples and data has demonstrated that the scale needed to achieve comprehensive datasets can be difficult to balance with the inclusion of local voices in governance of how such data is used moving forward [76]. Moreover, the infectious nature of pathogens can heighten equity concerns for pathogen repositories at a global scale, generating data-sharing concerns in contexts that historically have benefitted high-income countries over low- and middle-income countries (LMIC) [77,78].

## 3. AMR Is Distinct: Toward an ELSI of Precision Antimicrobial Stewardship

If we are to understand the toll of rising AMR, suffering and loss of life must be assessed in both quantitative and qualitative terms. In the U.S., an estimated 2.8 million people per year are infected with AMR pathogens [79]. In the worst cases, resistant infections are lethal. Mortality from specifically resistant infections is difficult to disentangle from sepsis more generally, and from mortality caused by related underlying comorbidities. However, deaths are estimated to be around 35,000 people per year nationally (caused by several common resistant pathogens) [79]. Burnham and colleagues more recently (2018) presented evidence that U.S. deaths due to resistant organisms are systematically undercounted, revising estimates for inpatient and outpatient deaths to be over 150,000 per year—more than four times the official CDC estimates [80].

Morbidity in and of itself also takes a toll. The experiences of patients and families grappling with resistant infections is also an area meriting new approaches to analysis, as burdens can be characterized through a variety of psychosocial lenses. Because AMR increases the length of stay for inpatients, it increases the burden of infection for patients and families while also increasing financial tolls on health systems. The broader national economic toll of AMR is sometimes estimated at $55 billion but redounds to individuals and those they financially support in a variety of ways. Rump and colleagues (2019) also reviewed the psychosocial toll of multidrug resistant infections, finding a variety of detriments including stigmatization, negative emotions, and interference with quality of care [81]. In U.S. hospitals, contact precautions that limit clinical communication and interaction are associated with increased levels of depression and distress [82,83]. Often, patients and caregivers continue to worry about exposing others and detail concerns of living with a resistant infection [84]. For example, patients diagnosed with Methicillin-resistant *Staphylococcus aureus* (MRSA) infections report mood disturbances; feelings of helplessness, shame, and dirtiness, as well as worries of susceptibility to repeat infections and hospitalization [84,85,86].

**Figure 1:**
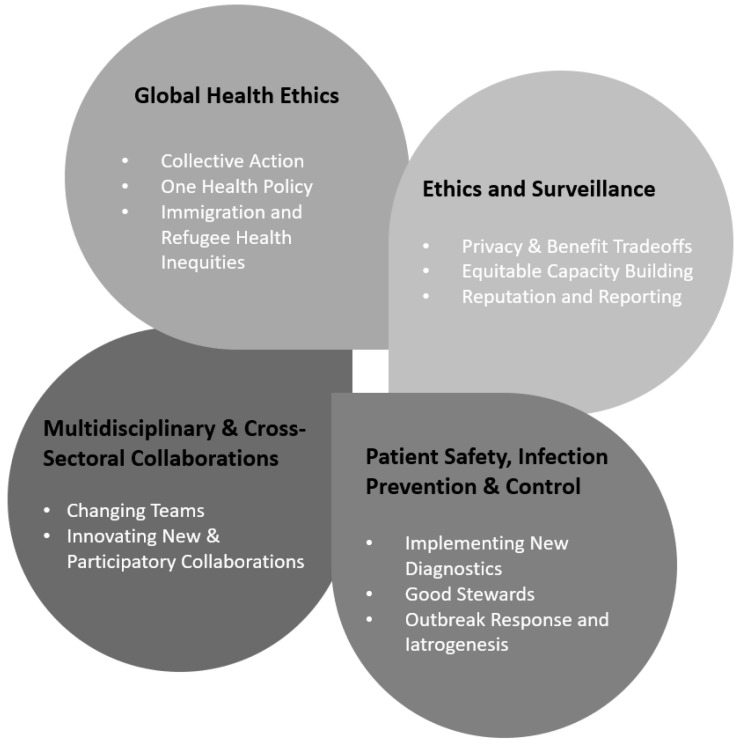
Distinct Domains for Ethical, Legal, and Social Implications Analysis of Antimicrobial Resistance.

Explicit ethical analysis concerning how we ought to be, think, and act about AMR is dispersed across multiple disciplines and distinct research dissemination networks. Recently, Frid-Nielsen and colleagues (2019) documented a lag in social science research about AMR, especially when compared to similar studies attending to other infectious diseases [28]. Meanwhile, Lu et al. (2020) used a co-citation analysis to improve the understanding of how AMR social science dissemination is dispersed across relevant areas of expertise, including medical, microbial, social science, and environmental health journals [27]. These efforts to explicate critical research gaps combine with a general rising call for greater international investment in AMR behavioral and social science research [5,6,7,8,9,10]. To this call, our analysis highlights how ELSI research helps refine health policy and ethical research priorities. In this section, we highlight four aspects of the AMR context that especially merit distinct consideration going forward (Figure 1).

### 3.1. Global Health Ethics

The first challenge of AMR ethics is to define it as a problem, including its ethical, political, and social dimensions. While ethical and social analyses are often turned to for decisional frameworks and problem-solving, their analytic power lies partly upstream, in the theoretical grounding that explicates and critically examines assumptions about how we conceive challenges in the first place [87]. At the heart of this definitional challenge is how we view and understand the relationships between humans and microbes [11,88]. Here, we review depictions of the rise of AMR in terms of collective action problems, One Health policy, global health justice, and immigration and refugee justice.

#### 3.1.1. Collective Action

Because of its inexact fit with public health emergencies, the rise of resistance is sometimes referred to as a “slowly emerging” or “silent” public health crisis [25,89]. Several scholars have noted the increasing moral characterization of AMR, including as a global collective action problem [19,24]. Littman and colleagues (2020) characterize AMR as a “superwicked problem” involving interdependencies that create obligations for organizational research, time sensitivity, and unclear ascription for responsibility [26]. A shift to social framing of the problem is sometimes offered as a counterpoint, moving away from excessive focus on technical, individual behavioral, or biomedical solutions [7,11].

#### 3.1.2. One Health Policy

The broadest framings of AMR acknowledge its cross-sectoral and ecological aspects, accompanied by corresponding normative proposed solutions [90,91]. For example, One Health frameworks entail ethical engagement with how responsibilities for human, animal, and environmental health interact, including questions on what multisectoral policy reform is needed in recognition of such interdependence [21]. In the U.S., agriculture accounts for approximately 65% of all AM use [92]; worldwide, AM use in animal agriculture is estimated at 93,000 tons [93]. There is considerable evidence that such use contributes to resistant infections [94] though causal relationships are still intensely debated [95]. A systematic review of psychosocial studies describes how veterinarians and farmers appreciate their role in generating AMR and the potential value of stewardship, but also identified low levels of concern and competing responsibilities to safeguard animal health [96]. Because genetic flow of resistance traits between animal companions, agricultural livestock, and humans continues to be difficult to causally untangle [97], which leads to a corresponding uncertainty about how to organize collective responsibility and action.

#### 3.1.3. Global Health Justice

Distinctly, AM stewardship raises many questions of global justice. Like other pathogens, resistant organisms spread regardless of national borders, escaping the levers of national policy [23,97]. The social and biomedical reality of interdependence laid bare by pathogens prompts ethical questions about what nations owe those who reside within their borders and to other nations [5]. Global health dynamics of shared need and disparate access to resources generate an imperative to respond [65]. Moreover, some LMIC contexts are still characterized by high mortality from infectious disease, which can drive wide availability of AMs [67]. Global health policy, too, has a long-recognized role in either sustaining or countering patterns of inequity [98].

#### 3.1.4. Immigration and Refugee Health Inequities

One aspect of global health and AMR that merits special attention is the disparate impact of resistant infections in underserved communities, especially refugee and migrant populations. For example, widespread resistance to isoniazid is more common in (often latent) cases of tuberculosis (TB) affecting foreign-born persons than those born inside U.S. borders [99]. This pattern is global, as incidence of TB in high income countries has rapidly decreased for the locally born, but it continues to disproportionately affect the foreign-born [99]. Phylogenetics is often defined as the use of genetics to study the evolutionary history and taxonomy of a group of organisms. This genomic advance is further offering evidence of the spread of *Staphylococcus aureus* infections—including MRSA—along migratory routes [100,101]. Such evidence is increasingly being used to inform health policy interventions, including surveillance and treatment programs situated around migration experiences [102]. When intertwined with rising social tension over migration, and likely increases of migration due to climate change or political unrest, phylogenetic and other molecular epidemiology findings have the potential to become deeply politicized. Among the ethical analyses needed is how to reduce inequitable health burdens of AMR in ways that actively undermine stigmatization. Genomic technologies also promise new advances for delivering narrow and tailored AMs in a timely manner. However, implementation must attend to unintentionally exacerbating inequity, especially in contexts characterized by xenophobia or racial animosity [103].

### 3.2. Precision Health Surveillance

Infectious disease surveillance is broader in scope than AM stewardship, in part because its goals are not limited to concerns about resistance and AM effectiveness. However, the two activities are programmatically combined. AM stewardship includes surveillance activities that encompass identifying how and where resistance is developing, predicting who is at risk for resistant infections, tracking down sources of resistant infections, and detecting emergent forms of resistance. In parallel, surveillance of prescribing practices seeks to identify patterns in who gives and who receives the most AMs, and how. The increasing use of AMR bioinformatics for surveillance purposes exemplifies the confluence of how genomic technologies facilitate new applications of the concept of public health surveillance. Surveillance is often defined as: (a) the systematic collection of pertinent data, (b) the orderly consolidation and evaluation of these data, and (c) the prompt dissemination to those who need to know and those in a position to take action [104]. Surveillance activities are known to generate privacy and trust concerns for affected communities, and genomic data can increase these by revealing geographic exposure history, including migration and incarceration [105]. For example, Jackson and colleagues (2019) found that Canadian stakeholders considered national TB surveillance to raise trust concerns between expert and lay communities, as well as between different experts [106]. Elsewhere, human immunodeficiency virus (HIV) genomic surveillance is receiving bioethicists attention for raising concerns about consent for data reuse, privacy, and potential criminal ramifications [107,108].

#### 3.2.1. Equitable Capacity Building

With horizontal as well as vertical inheritance in the mix, phylogenetics provides insights into how resistance emerges and moves across both microbial and host populations. COVID-19 has demonstrated both the value of phylogenetic analysis and disparate geographic differences in pathogen surveillance capacity. The first global collaborative effort to develop shared AMR surveillance standards was launched with the 2015 Global Antimicrobial Resistance Surveillance System (GLASS) [109]. Global health surveillance and reporting to GLASS involves a variety of voluntary national participation and incentives to build out the infrastructure needed, including building capability between national systems and GLASS. Among other ethical traditions, human rights frameworks are well-recognized for supplying political, humanitarian, and ethical rationales to accompany the logistical imperatives of global health program building [110]. Domestically, public health obligations to address health disparities entails attending to the distribution of surveillance capacity across U.S. regions, states, and localities [111].

#### 3.2.2. Reputation and Reporting

Locally and regionally, advances in genomic surveillance have the ability to complement other sources of data, including community or hospital antibiograms [112]. However, the difficulty of treating resistant infections can heighten the stakes of such population health data. Reporting can generate political concerns for public and hospital leaders, including revelation of health systems weaknesses, mismanagement, or poor performance in comparison to other national or regional comparators. For example, outbreaks of MRSA in the National Health Service of the United Kingdom are deeply politicized, including during elections [113,114]. Leaders across the world have been known to withhold public health data out of fear for local political blowback and concerns that outbreaks will damage local economies. AMR global and hospital surveillance systems are thereby situated in a complex sociopolitical context in which health policy must motivate transparency while also addressing the reputational concerns of reporting entities [115].

### 3.3. Ethics, Patient Safety, and Precision Infection Prevention and Control

Genomic advances in precision infectious disease hold out the promise of dissolving some of the ethical challenges of AMR. Most of these ethical tensions are conflicts between clinicians’ fiduciary obligations to current patients and stewardship obligations to conserve AM usefulness for future patients. Current efforts to improve AM prescribing practices depend, in part, upon more efficient infectious disease diagnostics. The promise of “precision metagenomics” is to shorten the time it takes to gain crucial information about hosts, genes, and microbes, from days or weeks, currently, to mere hours [3]. Precision methods can also offer hope of improved and tailored treatment [116]. By getting exact AM susceptibility information and new tools into the hands of treating clinicians faster, precision approaches seek expanded opportunities to deliver more tailored infectious treatments earlier or even as a first line of treatment.

#### 3.3.1. Implementing New Diagnostics

Increasingly rapid diagnostic technologies (RDTs) illustrate the potential of technological advances. For example, Bookstaver and colleagues (2017) report that including RDTs as part of an interventional bundle decreased time to de-escalation of AM therapy [117]. However, one implementation challenge with RDTs is that stewardship team members can lack familiarity with their benefits [118]. Further complicating utilization of RDTs are the dynamics of sufficiency and clinical validity. For example, confirming rifampicin resistance can require traditional susceptibility testing, rendering RDTs an additional expense that is especially prohibitive in LMICs [119]. Meanwhile, in malaria, the uptake and adherence to RDTs is highly context-dependent [120]. When de-escalation is possible, RDTs rapidly allow for infectious disease consultation and can lead to a prescription of a narrower therapy, reduced duration of treatment, or minimize risks of adverse events [121,122].

However, the ability to tailor treatment to an infection is widely variable depending on the sensitivity and specificity of the test, the turnaround time, the nature of the infection, the impact of comorbidities, and the availability of treatment options. The promise of better diagnostics cannot be fulfilled without access to subsequent care. On top of the implementation barriers for diagnostics, crucial bottlenecks continue to remain in drug development: a challenge encountered elsewhere in precision medicine [123] and that can present new ethical dilemmas in AMR [124]. The well-documented economic obstacles to establishing a reliable AM drug development pipeline is increasingly taking on social and moral significance [125]. At a time of increasing public distrust of pharmaceutical companies, policy attempts to overcome longstanding dynamics of AM market failures manifest very differently across national contexts [126,127].

#### 3.3.2. Good Stewards

Among the many challenges of AM stewardship, one possible framing is in terms of intergenerational justice: how to appropriately balance the wellbeing of current and future patients [128]. Variably termed appropriate, prudential, or rational use of AMs, stewardship is also often understood as a classic challenge of implementation science; evidenced-based standards of AM use are established, but integrating this knowledge into practice is no simple matter [129]. Behavior change is notoriously challenging for humans, including experts. Successful interventions require addressing all the elements of behavior, from systems to culture, conscientious decision-making to ingrained assumptions. While advances in pathogen genomics might facilitate behavior change, individual choices are socially situated and contextually determined. Implementing new tests or technologies takes place within extant concerns about liability, reputation, clinical autonomy, or fairness in workplace evaluation [130,131,132].

#### 3.3.3. Outbreak Response and Iatrogenesis

Whole genome sequencing is also increasingly facilitating rapid hospital outbreak response [133,134]. For example, Angletti et al. (2015) reported the advantages of the new genomic methods and noted how genomic results pointed toward a portable X-ray as a possible source of a resistant pathogen hospital outbreak [135]. While such advances enable harm reduction, the implementation of new genomic technologies require integrating into an existing knowledge base for generating cultures of safety, including medical error [132,136]. Medical ethics literature has a well-established discourse on medical error, including on the need for analysis to move to the systems level, and to support responsible disclosure [137,138,139,140]. Systems approaches have attended to unproductive silencing [141], and the collective nature of cultures of safety [142]. Other aspects of health safety that could merit ELSI analysis include: the second victim phenomenon, leadership accountability, health systems billing structures, respect and psychological safety that allow staff to admit error, and ensuring patient-centered care that attends to the lived experiences of those affected by hospital-acquired infections.

### 3.4. Multidisciplinary and Cross-Sectoral Collaborations

Nationally, AM stewardship program (ASP) teams are clinically assembled to reflect the multidisciplinary complexity of AM policy and practice across health systems. Pragmatically, stewardship activities are also undertaken by staff members who are available to shoulder additional responsibilities. Current guidelines prioritize advanced stewardship expertise, achieved either through leadership by a trained infectious diseases (ID) physician, or codirection by an ID physician and a trained clinical pharmacist [143]. Stewardship teams can also include expertise from clinical microbiology, infection prevention and control, and nursing. Nurses play a crucial role, both as members of stewardship teams and when working in conjunction with them [144]. Meanwhile, pharmacists’ roles vary dramatically depending on policy and resources [145].

#### 3.4.1. Changing Teams

Advances in pathogen genomics will be situated within the team dynamics of IPC, ASP, and research projects [1]. The relational corollary to questions of integrating new forms of knowledge is achieving change among knowledge-holders. As pathogen genomics advance, what happens when existing roles (e.g., that of microbiologists) are expanded or new forms of expertise (e.g., computational biology) are added to the team? Van Goethem and colleagues (2020) anticipate this challenge, describing differing attitudes of pathogen genomics data providers and end-users in Belgium, capturing both existing knowledge gaps and accompanying gulfs in expectations for clinical and population health impact [146]. The study of attitudes and behavior, both individual and collective, will be needed to help IPC and ASP teams expand.

#### 3.4.2. Innovating New and Participatory Collaborations

From an ELSI perspective, ASP teams also generate a set of questions that engage with role morality, or the enculturation process that orients professionals to distinct values [147], and their interprofessional interactions [148]. Moreover, One Health policy questions will challenge team science to bridge agricultural and health settings—with some lessons to be drawn from related but distinct non-human ELSI research, such as genetically modified plants or mosquitos [9,149]. In this space, there is great potential for innovation in both team science research design and community engagement [150,151,152].

## 4. Conclusions: Advancing an ELSI Precision Stewardship Agenda

Recently, several calls to address the rise of resistant pathogens have emphasized the importance of social science to AM stewardship. AMR is a phenomenon whose causal factors range from the molecular to that of global health policy. As such, it requires solutions that span from microbiology to the most macro-policy levels. In this article, we consider how the longstanding U.S. NHGRI ELSI Program suggests one potential approach to organizing a social science and policy research agenda.

The field of ELSI scholarship developed alongside the Human Genome Project. Decades of ELSI research explores how the implementation and use of genetics are shaped by, and in turn shape, U.S. policy, social dynamics, and values. Though long recognized, the crucial role of genetics in tackling the problem of AMR is increasingly informing new approaches to AM stewardship. Next generation sequencing has accelerated the understanding of human and pathogen genomes alike. Resistance develops through natural selection after exposure to AMs, creating environments that encourage the development and transmission of resistance genes. Rapid molecular identification methods include the use of genetic and other molecular techniques to speed the time of test results, promising clinicians new and improved diagnostics and timely information to improve their prescribing practices.

The falling cost of microbial genetics has produced a companion rise of scientific publications in pathogen genomics, although the implementation of these technologies in clinical microbiology and laboratory medicine is not without its challenges. As in human genetics, a corollary ELSI agenda accompanying pathogen genetics invites multidisciplinary scholarship and innovation. New forms of collaboration provide an opportunity for novel approaches in AM stewardship.

## Figures and Tables

**Table 1:** Recent Work Reframing The Challenge of Antimicrobial Resistance.

Framing of AMR	Source	Title	Quote
Stewardship	Broom et al. 2020. [11]	Antimicrobial resistance as a problem of values? Views from three continents	*“The move from de-contextualised information (data) to social action is highly dependent upon various forms of social relations, power dynamics, and the institutionalisation of professional and political practice.”*
Global health justice	Hoffman et al. 2015. [23]	An international legal framework to address antimicrobial resistance	*“To avert millions of deaths caused by treatable infections, access to antimicrobials should be scaled-up for the many people worldwide who cannot obtain or afford such drugs.”*
Collective action problem	Giubilini 2019. [24]	Antibiotic resistance as a tragedy of the commons: An ethical argument for a tax on antibiotic use in humans	*“Antibiotic effectiveness is a common good, or a common pool resource. These types of goods are defined, among other things, by the fact that each individual who enjoys them contributes to their erosion.”*
Slowly emerging epidemic	Viens and Littmann. 2015. [25]	Is Antimicrobial Resistance a Slowly Emerging Disaster?	*“Whether terms such as disaster or pandemic are contested concepts or reflect different health issue prioritizations, how we define and use technical terms matters. We should be very wary of people who throw such terms around so capriciously as synonyms for public health problems that are important, urgent or serious.”*
One Health policy	Antoine-Moussiax et al. 2019. [21]	The good, the bad and the ugly: framing debates on nature in a One Health community	*“While each positionally objective view may be useful, when considered separately, they may lead to mistakes due to the biased perspective of the position. The main issue is not to assess the impact of logically equivalent (i.e., having the same truth-value that is, being verified by the same facts) framings on decision-making, but to deal with descriptions having different truth-values (i.e., being verified by different facts and actors).”*
Wicked problem	Littmann, Viens & Silva. 2020. [26]	The Super-Wicked Problem of Antimicrobial Resistance	*“To put it another way, through framing AMR as a super-wicked problem, we not only acknowledge that previous approaches are unlikely to be sustainable in the long run, but we are also forced to ask what sort of values and norms could justify new policy options that would not only be effective but also ethical. This is all the more relevant because understanding AMR as more than a scientific or technical issue is a relatively new perspective.”*

**Table 2:** Shared Domains for ELSI of Human and Resistance Genetics.

Shared Domains	ELSI and Human Genetics	ELSI and Resistance Genetics
Understanding and attitudes	Genetic determinism	Views on immunity, infections
	Familiarity and expectations	Implementation barriers and facilitators
	Genetic literacy	Cross-sectional affected stakeholders
	Disclosing to third parties	Communicability
Health equity	Insurance discrimination	Global health policy
	Information and empowerment	Access and excess interrelatedness
	Eugenics	Disparities of disease burden
	Commercialization	Drug-development pipeline
	Race, essentialism, genetics	Laboratory and research capacity
Data governance	Privacy and identifiability	Surveillance and privacy
	Data sharing	Global health collaborations
	Reciprocity	Local trust
	Broad consent	Data for action
	Utilization and sustainability	

## Data Availability

Not applicable.
